# Alain G. Cribier: 1945-2024

**DOI:** 10.21542/gcsp.2024.20

**Published:** 2024-04-20

**Authors:** Magdi Yacoub

**Affiliations:** Imperial College, London, UK & Aswan Heart Centre, Aswan, Egypt

**Alain Cribier, the French cardiologist who performed the world’s first transcatheter aortic valve replacement (TAVR) and has been responsible for a number of other world firsts, died on 16th February 2024 in Rouen, France. He was 79.**


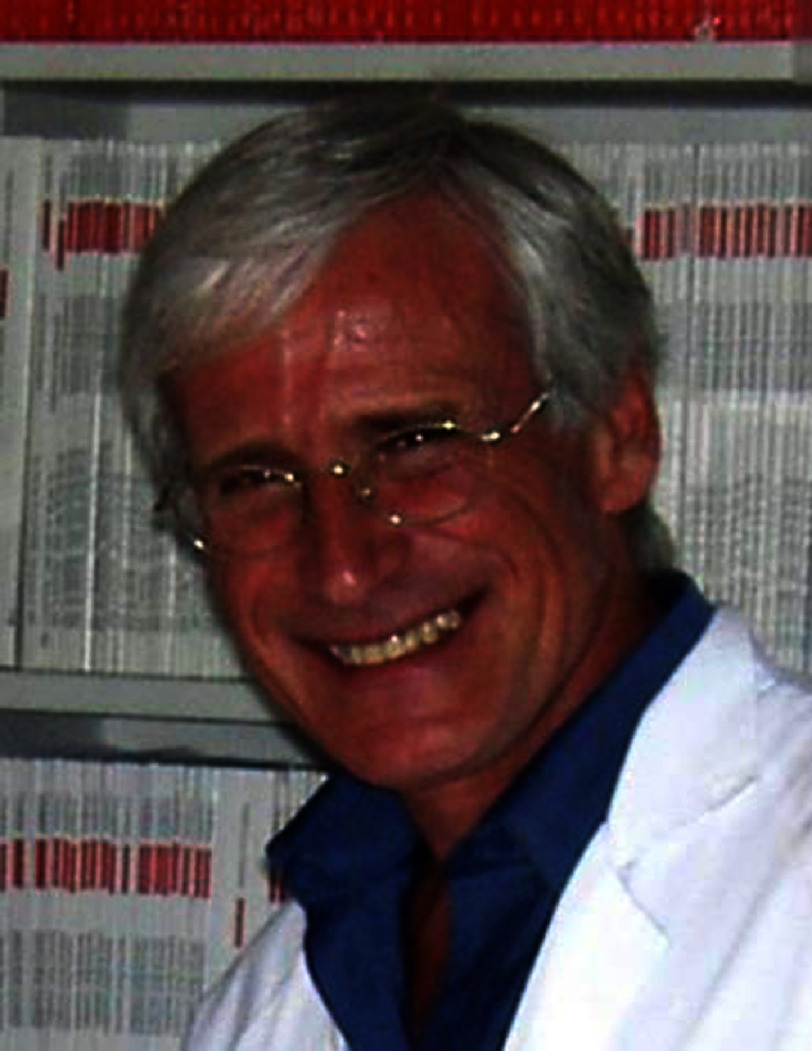


**Alain Cribier**^1^ 1Reproduced under CC BY 4.0 license from https://doi.org/10.1016/j. jscai.2024.101860


Cribier successfully fulfilled a wish he had held since childhood to become a doctor. Speaking to *Cardiovascular News* in 2003 he recounted: “*As far as I remember, my wish has always been to become a doctor. I was certainly influenced by some of my early reading, such as the books of Cronin, Duhamel and Schweitzer in which I easily identified myself with some outstanding physicians who dedicated their whole life to the care of other people”.* Indeed Albert Schweitzer continued to be an influence on Cribier throughout his early career.

Criber was born in Paris on 25th January 1945 to Marie-Thérèse and Camille Cribier and attended Charlemagne-Lycée for his high school education and baccalaureate. However, his early desire to become a doctor was rivalled by another of his passions, music. He was, even at an early age, an incredibly gifted musician and considered becoming a concert pianist. His love of Rachmaninov is obvious from the recitals on his YouTube channel^2^. 2
https://www.youtube.com/ @alaincribier4751



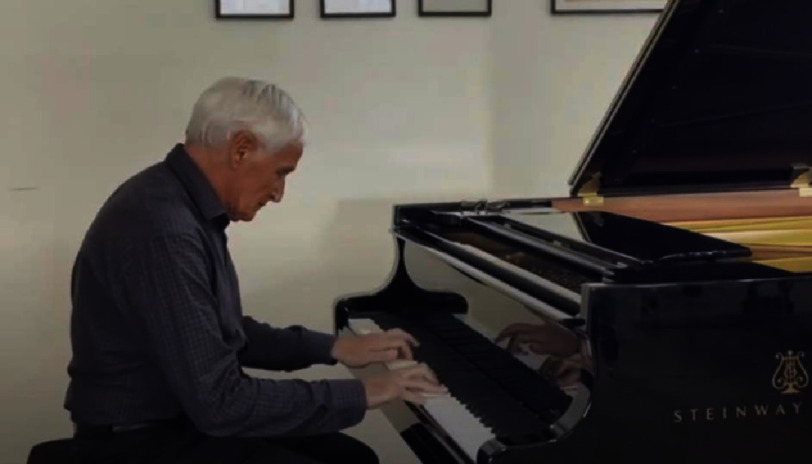


**Alain Cribier indulging in his second love of the piano.**

Cribier’s medical training started at University of Paris in 1962, where he spent his spare time working as an instrumentalist in the operative rooms for Prof Charles Dubost—a man for whom he had ‘the deepest admiration’.

He became entranced by the promise the future held for cardiac surgery, especially the more invasive procedures which were emerging at the time (cardiac catheterisations, coronary arteriographies, and early CABG and valve replacement). In fact his experience of witnessing a cardiac catheterisation at Hospital Tenon in Paris in the early 1970s convinced him to pursue a specialization in medical cardiology.

In 1972, after receiving his certification in cardiology (as well as a diploma in Law and Health Economics), he decided to do his residency at Charles Nicolle University Hospital in Rouen, France—joining the Department of Cardiology headed by Professor Brice Letac. Letac was another person destined to have a long-lasting influence on Cribier’s career, providing advice and encouragement throughout their 20-year collaboration.

In 1976, having completed his Master’s thesis on “Left ventricular angiography for the evaluation of left ventricular function”, he went to work abroad for a year and found himself at Cedars Sinai Hospital in Los Angeles under the direction of Dr Jeremy Swan and Dr Willaim Ganz. Cribier would later say that these two doctors “opened my mind to the concept of research”, which was timely as he completed his Medical thesis on “Hemodynamic study of idiopathic cardiomyopathies; correlation with clinical data” under their supervision in 1977.


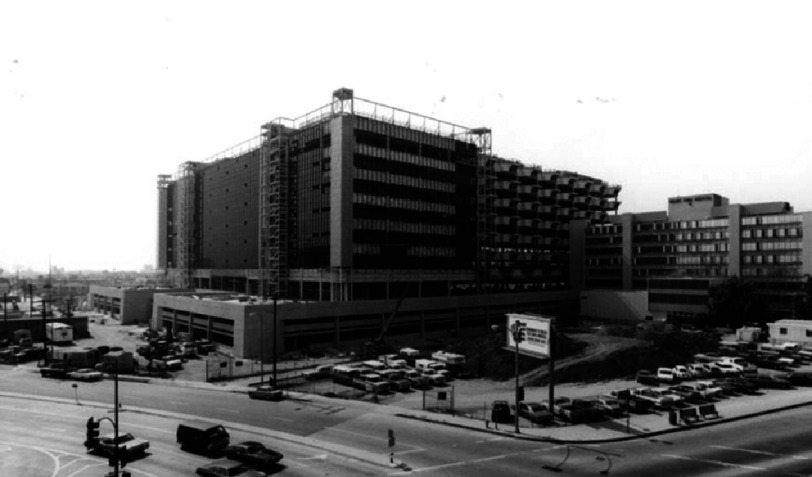


**Cedars-Sinai Hospital in Los Angeles in the mid-1970s.**

He returned to Rouen in 1977 and spent the majority of his life from that point in the city. In 1983 he was offered the position of Professor of Medicine and Director of the Cath-Lab at Charles Nicolle Hospital, University of Rouen. This new position offered Cribier full access to patients, university teaching, and research and allowed him to start considering new interventions in cardiology.

The first of many world firsts saw Cribier perform the first balloon valvuloplasty for calcific aortic stenosis in 1986.^3^ 3Cribier A, Savin T, Saoudi N, Rocha P, Berland J, Letac B. Percutaneous transluminal valvuloplasty of acquired aortic stenosis in elderly patients: an alternative to valve replacement?. *Lancet*. 1986;1(8472):63-67. doi:10.1016/s0140-6736(86)90716-6This percutaneous procedure, *via* a femoral vein, opened calcified valve leaflets and improved blood flow through the valve. It offered positive short-term relief, but mid-term restenosis rates were high and spurred Cribier on to find a better technique.

He settled on the idea of a percutaneous heart valve as a route to better long-term outcomes, but it would take many years for his vision to become a reality. Biomedical companies were unconvinced that the concept could work. As a result, Cribier and others established Percutaneous Valve Technologies, a start-up dedicated to the development of his vision of a percutaneous aortic valve replacement.

After a number of a years of research, design, iteration, and animal trials, the day of 16th April 2002 will be a landmark date in cardiology, as it was on this date that Cribier performed the first transcatheter aortic valve replacement (TAVR) in the world. The patient, a 57-year-old male, suffered a relapse from a previous balloon valvuloplasty but within a few hours of the TAVR procedure, was well enough to get out of bed. “The patient resuscitated on the table. Before that, he was practically dead; he had had several episodes of cardiac arrest before entering the cath lab. Immediately after the valve was implanted, the colour came back to his face, and he was speaking. We could observe a true resuscitation!”

In the intervening years, TAVR has gradually matured, established itself as an effective, minimally-invasive treatment, and changed the landscape of interventional cardiology, especially for those patients who would otherwise be considered ‘inoperable’.

But Cribier offered the world of cardiology much more than TAVR. While PVT was developing the first percutaneous valve replacement, he also developed the metallic commissurotome, an instrument conceived to improve the cost-efficacy of mitral valvuloplasty. The tool became immensely popular in developing countries where mitral stenosis remains endemic. As a result, Cribier strengthened his ties with many countries in Asia, but particularly India. He subsequently co-founded the Indo-French Foundation of interventional Cardiology in 1996.

Since 2013, and having retired from Charles Nicolle Hospital, Alain Cribier and Hélène Eltchaninoff ran the MTC (Medical Training Center) in Rouen, a multidisciplinary center dedicated to teaching medicine through simulation, video-conferences and training between surgeons, physicians and experts. TAVR is one of the most popular specialities they offer training in.

He will be remembered for his humility, his energy, and his perseverance - on top of his inventiveness and openness. He was always available to those who sought his opinion, and offered his insights in an encouraging manner.

He had a profound impact on countless lives and families, and the world of cardiology has lost not only a caring doctor, but also a friend and visionary.


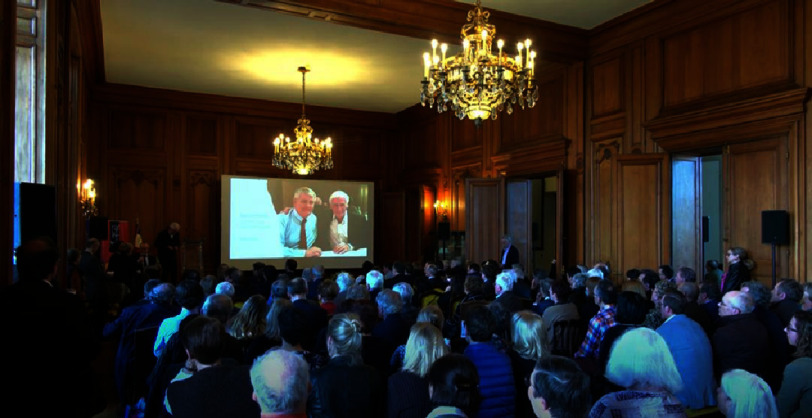


**Readers may be interested to know that a 3-hour celebration of Cribier’s life and contributions to cardiology, held in Rouen town hall, is available on YouTube: https://www.youtube.com/watch?v=3rzjJUgIAcI**

